# Observational study on calf carcasses in 2 processing plants for animal byproducts in Germany

**DOI:** 10.3168/jdsc.2024-0687

**Published:** 2025-03-03

**Authors:** M. Sickinger, A. Wehrend

**Affiliations:** 1 Clinic for Ruminants and Herd Health Management, Justus Liebig University Giessen, Giessen, Germany 35392; 2 Veterinary Clinic for Reproductive Medicine and Neonatology, Justus Liebig University Giessen, Giessen, Germany 35392

## Abstract

In the dairy industry, surplus calves have been reported to be especially prone to poor welfare on farms and to inappropriate killing of male calves for economic reasons. Therefore, this study aimed to examine calf carcasses for evidence of inappropriate killing and diseases that may have caused prolonged suffering and unnecessary pain in the course of their lives. Our study was conducted during March 2022 and from April to May 2023 in 2 animal byproduct processing plants, where we carried out external inspections of the carcasses to record the sex of the animals and check for the presence of identification marks. We paid particular attention to findings relevant to animal welfare that suggested improper killing or unnecessary suffering and pain in the animals. The examinations included observations on the skinned carcasses (in plant 1), as well as on carcasses in the blanket, the opening of the trachea, and the carpal and tarsal joints (i.e., partial necropsy). In total, 981 calves from dairy cattle and beef cattle herds were examined on 19 delivery days. This encompassed 450 calves at the first location and 531 at the second location. In total, 515 calves were male and 465 were female. The sex of one animal could not be determined because of the nutritional trace on the carcass. Ear tags were not inserted in 588 calves. Five of the 588 calves initially had ear tags. Ear tags were detected in 393 calves. At partial necropsy, 18 animals were diagnosed with profound emaciation. Additionally, arthritis existed in 9 animals. Chronic diseases other than arthritis were observed in 27 (2.8%) of 981 animals. Arthromyodysplastic syndrome was diagnosed in 26 animals. Fractures were found in 38 animals, of which only 3 cases occurred antemortem. The remaining 35 cases of fracture occurred postmortem as a result of transportation. No animal had signs of improper obstetric care. Amniotic fluid was detected in the lumen of the trachea of 122 stillborn calves (i.e., death sub natu). In contrast to the situation reported in pigs, no evidence existed in the 2 animal byproduct processing plants investigated for calf carcasses that inappropriate killing and diseases leading to prolonged suffering and unnecessary pain before death represented systemic problems. No fundamental need for routine testing of animal carcasses existed in either animal byproduct processing plant.

## Highlights

• The dairy industry produces surplus or nonreplacement calves with low economic value.
• Poor animal welfare conditions in these male calves have been reported.
• Our study aimed at exploring animal welfare and care in these calves.
• Male calves were not overrepresented, with no signs of prolonged suffering.
• Routine carcass testing is not mandatory at the 2 examined processing plants.

## Graphical Abstract

**Figure:**
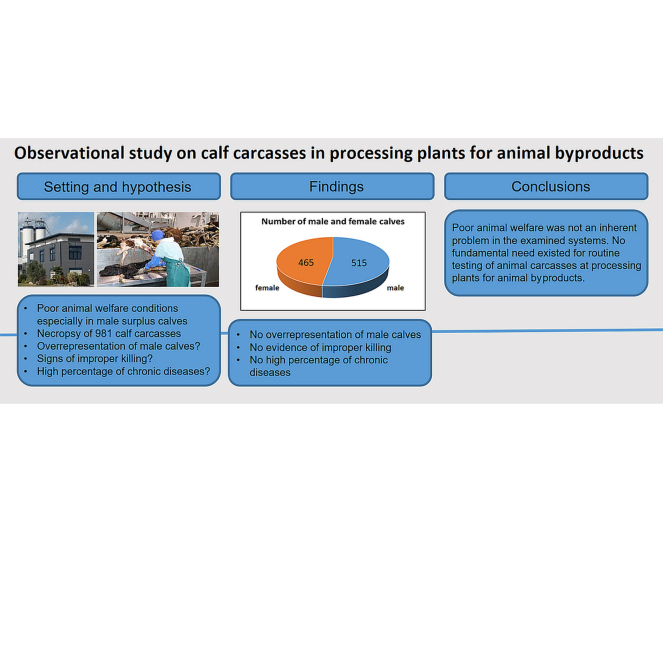
**Summary:** The dairy industry inevitably produces surplus calves that are of low economic value and prone to poor animal welfare and care. Illegal killing of calves or withholding of medical care has been reported for dairy and beef breed calves. Therefore, we examined all calf carcasses up to ~6 mo of age in 2 processing plants for animal byproducts in Germany. Our examinations were conducted during March 2022 and from April to May 2023, focusing on the male-to-female calf ratio and identifying indicators of prolonged suffering, such as arthritis, chronic bronchopneumonia, and emaciation, as well as signs of poor animal welfare standards at the farms of origin.

To maintain high milk yield in dairy cows, each cow typically gives birth to one calf per year, resulting in ∼22 million calves per year in the European Union and 470,000 calves in Canada (Zinke, 2020; Reed et al., 2022; Eurostat, 2024). Comparable high numbers of calves are produced worldwide (Bolton et al., 2024), exceeding the needs of dairy operations and subsequently resulting in the so-called surplus or nonreplacement calves. Depending on the regional dominating dairy production system, these primarily male surplus calves are typically either culled early on the farm or fattened for a period before being slaughtered for meat production (Boyle and Mee, 2021; Bolton et al., 2024; Maggard et al., 2024). Because of their low economic value, these surplus calves are especially prone to poor welfare and low-intensity medical care while on dairy farms (Boyle and Mee, 2021; Maggard et al., 2024). Additionally, not only dairy surplus calves, but also crossbred calves destined for early-life slaughter face the risk of impaired welfare on farms, during transport, and at abattoirs (Bolton et al., 2024). Depending on the production system, even suckling or beef calves that are raised for meat production face the risk of low animal welfare conditions (Leruste et al., 2014), though the risk is considered lower than that in dairy surplus calves (Mandel et al., 2022). Therefore, the first attempts to monitor general animal-based measures or animal-based indicators in slaughterhouses to improve on-farm welfare have been reported (Boyle and Mee, 2021; Nielsen et al., 2023). These ante- and postmortem indicators for poor on-farm, in-transit, and at-abattoir welfare in calves aged <1 mo, include umbilical maturity, nutritional status, behavior, signs of disease, lameness, injuries, arthritis, peritonitis, and pneumonia (Boyle and Mee, 2021). Application of such ante- and postmortem examination schemes in farm animals demonstrated that >10% of animals that die or are culled on farms are prone to poor animal welfare conditions causing prolonged suffering (Große Beilage, 2017; Große Beilage et al., 2021). This factor emphasizes that animal-based measures or animal-based indicators at the abattoir level may underestimate the prevalence of chronic diseases, high mortality rates, and improper killing of male dairy and beef calves on farms because these animals do not reach abattoirs, but are instead sent to processing plants for animal byproducts. These processing plants are responsible for the hygienic removal of carcasses of dead animals from farms and private holdings, remnants of slaughtering, and from the food production industry, as well as for the elimination of carcasses in cases of epizootic diseases. Therefore, the aim of our study was to investigate the condition of all delivered calf carcasses (dairy and beef breeds), with special attention to the ratio of male to female calves and all signs that may be related to prolonged disease (i.e., arthritis, chronic bronchopneumonia, and emaciation) or low animal welfare standards on the farm of origin.

The investigations were conducted from March 9 to March 31, 2022, and from April 20 to May 15, 2023, on 19 delivery days in 2 processing plants for animal byproducts. The plants were in the west (plant 1) and east (plant 2) of Germany. The catchment area of plant 1 primarily represented small family-run farms, whereas that of plant 2 represented large-scale farms. Both catchment areas had a radius of ∼130 km. During delivery, cattle up to an estimated age of 6 mo were sorted by the employees of the processing plants and prepared separately for assessment (Figure 1A). Approval of the Animal Ethics Committee was not required because the study was performed with deceased animals.

**Figure 1:**
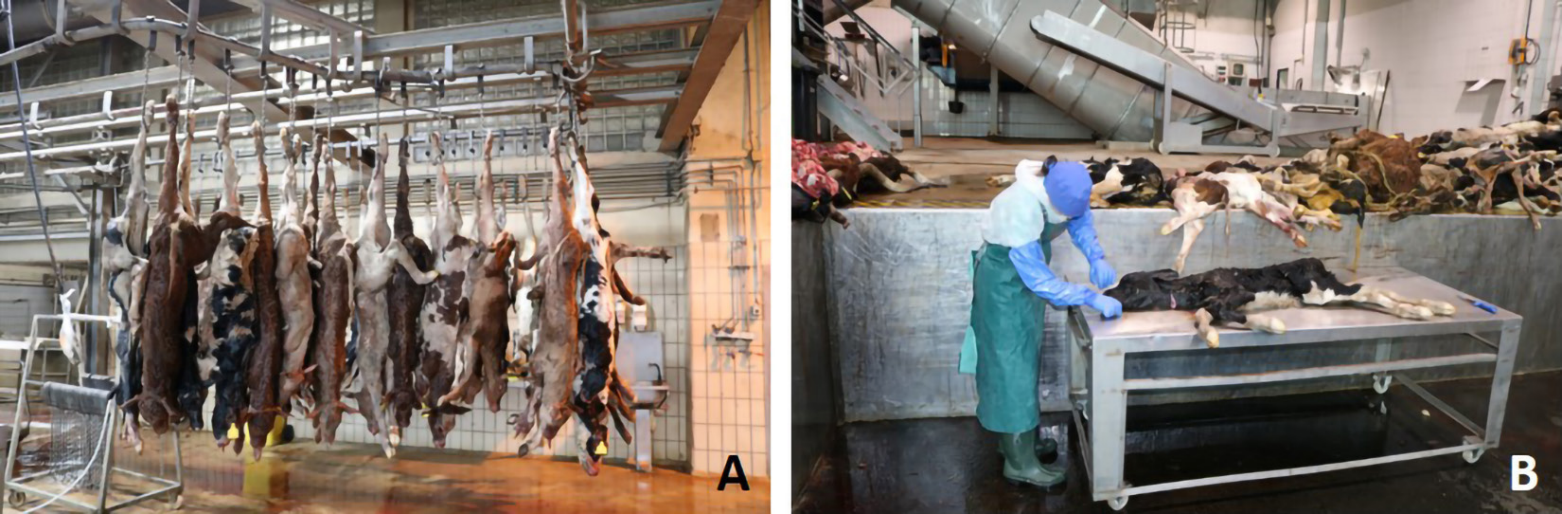
(A) Calves prepared for examination in a hanging position at plant 1. (B) Performance of partial necropsy in plant 2.

Depending on technical prerequisites, the animals were either placed on a table for examination or their carcasses were inspected while hanging on hooks (Figure 1B). In the plant in which an assessment on a hanging animal could be carried out, the carcasses were skinned as part of the processing. Therefore, the dissection of the carcasses included an assessment of the skinned carcasses.

The external examination was conducted by the authors (experts in the field of ruminant internal medicine, surgery, and reproductive and neonatal medicine) and included breed assignment, sex determination, checking for the presence of an ear tag, dental findings, and assessment of the dental arch to estimate the age of the animal. In addition, the degree of soiling; condition of the coat, head, eyes, mouth cavity, navel, anus, limbs, and joints; nutritional status; and presence of wounds were recorded. Crown–rump length and chest circumference were also recorded during holding, which enabled the hanging assessment of the carcasses. A more detailed assessment consisted of opening the carpal and tarsal joints and the trachea. In suspected cases (e.g., swelling, abscesses), additional joints, such as the knee, were opened. The trachea was opened to evaluate whether foreign material had been forced into it, whether tracheal discharge existed, and whether amniotic fluid was detectable. Calves with amniotic fluid in the trachea were considered stillborn.

The ratio of male to female calves in both processing plants was statistically compared, using the chi-squared test for equal proportions. The significance level was set at *P* = 0.05.

In total, 981 calf carcasses were examined during the study period. These animals were allocated into 450 (x̅ ± s: 50 ± 11.8) calves at plant 1 and 531 (x̅ ± s: 53.1 ± 11.8) calves at plant 2. Plant 1 was visited 9 times and plant 2 was visited 10 times (Table 1). To evaluate the general welfare conditions of calves within the catchment areas of the 2 plants, all calves that had been delivered to both locations were examined. Breed distribution showed that 63.5% were dairy breeds and 36.5% were classic beef breeds, with Simmental calves classified as beef breeds despite their dual-purpose use for both dairy and beef production. A detailed overview of breed affiliation is provided in Table 2.

**Table 1:** Number of male and female calves delivered to plants 1 and 2 per day

Plant	Day									
	1	2	3	4	5	6	7	8	9	10
1										
Male	30	22	17	25	31	17	37	29	23	—1
Female	25	28	17	19	23	18	35	33	20	—1
2										
Male	29	26	33	29	27	17	42	24	27	30
Female	25	23	28	36	19	18	33	20	19	27

1 Plant 1 was not visited at d 10.

**Table 2:** Overview of breed affiliations of examined calves; total numbers and percentages in reference to the total number of examined calves are given

Breed, n (%)	Plant 1	Plant 2
Aberdeen Angus	4 (0.4)	13 (1.3)
Belgian Blue	2 (0.2)	0
Brown Swiss	1 (0.1)	0
Charolais	13 (1.3)	8 (0.8)
Galloway	1 (0.1)	1 (0.1)
Hereford	0	2 (0.2)
Holstein Friesian	218 (22.2)	293 (29.9)
Holstein Friesian crossbreds	6 (0.6)	27 (2.8)
Limousin	107 (10.9)	39 (4.0)
Red Holstein	51 (5.2)	27 (2.8)
Scottish Highland	2 (0.2)	0
Simmental	26 (2.7)	105 (10.7)
Simmental crossbreds	7 (0.7)	2 (0.2)
Other	12 (1.2)	13 (1.3)

The age of the animals was estimated according to the presence and shape of the teeth alignment as well as the degree of gingival coverage of the teeth. Calves with overlapping incisors and gingiva covering the teeth were considered to be 2 to 3 wk old, whereas those with aligned incisors and retracted gingiva were classified as older than 4 wk (Stöber, 1990). Based on this classification, 457 calves were 2 to 3 wk old, 45 aged 3 to 4 wk, and 472 calves were deemed older than 4 wk. Age estimation was not possible in 7 animals due to missing lower jaws.

The sex distribution of the calves did not differ, with 231 (x̅ ± s: 25.7 ± 6.3) male calves and 218 (x̅ ± s: 24.2 ± 6.2) female calves at site 1 (*P* = 0.54), and 284 (x̅ ± s: 28.4 ± 6.4) male calves and 247 (x̅ ± s: 24.7 ± 6.3) female calves at site 2 (*P* = 0.11; Table 1).

When examining the calves at plant 1, determining the sex of one animal was no longer possible because of nutritional traces on the carcass (genital organs were missing due to signs of erosion where wild animals had eaten away at the carcass). The crown–rump lengths of the calves examined at plant 1 averaged 86.1 ± 7.8 cm (minimum [**Min**]: 65 cm; maximum [**Max**]: 114 cm), and the mean chest circumference was 72.2 ± 7.5 cm (Min: 53 cm; Max: 105 cm).

The number of calves with ear tags was 393 (40.1%). A total of 588 (59.9%) calves at both locations had no ear tags inserted, or the tags were no longer detectable. In calves where the ear tag was no longer detectable, the punched holes of the ear tags remained visible (n = 3), or the ear tags were torn out (n = 2). Additionally, 382 calves out of all calves aged older than 3 wk (n = 517) had no ear tags (73.9%).

Among the 981 calves, at partial necropsy, 18 (1.8%) were profoundly emaciated, indicating a prolonged duration of disease or malnutrition. Arthritis was present in a total of 9 animals (0.9%), namely inflammation of the carpal joints in 6 animals, tarsitis in 2 animals, and right-sided gonitis in one calf. Chronic diseases other than arthritis were deemed present in 27 (2.8%) animals, showing signs of emaciation, bronchopneumonia, or diarrhea.

Arthromyodysplasia syndrome was diagnosed in 26 (2.7%) animals, and fractures were found in 38 animals (3.9%), of which only 3 cases occurred intra vitam. The remaining 35 cases occurred postmortem as a result of on-farm handling after death or transportation of the carcasses to the processing plants. Fractures were regarded as intra vitam occurrences if the bone-surrounding tissue showed signs of hematoma or swelling. No signs of improper obstetric care were observed in any animal.

Tracheal content consisted of amniotic fluid within the trachea of 122 stillborn calves (i.e., death sub natu). Purulent tracheal discharge was present in 61 (6.2%) animals, and no signs of forced suffocation (e.g., expanding foam, foreign bodies) could be revealed.

The dairy industry produces a large number of calves that exceed their operational needs (Maggard et al., 2024). Male calves are overrepresented among the so-called surplus calves because only female calves may serve as future replacement dairy cows (Creutzinger et al., 2021; Reed et al., 2022). Because of the lower value of male calves in the dairy industry, they are particularly prone to reduced welfare and care. In addition, social awareness of animal welfare has increased over the past decades, leading to various studies focusing on animal-based indicators at farm sites, during transportation, or at abattoirs (Boyle and Mee, 2021; Creutzinger et al., 2021; Hayer et al., 2021). Although numerous studies have concentrated on the general welfare of calves (Nielsen et al., 2023; Maggard et al., 2024), examinations of female dairy calves have exceeded those of male dairy calves (Godden, 2008; Urie et al., 2018; Shivley et al., 2019; Reed et al., 2022). This factor again represents the higher economic value of female dairy calves. Sex differences in management measures include early and sufficient volumes of high quality colostrum, dehorning practices, and pain management (Shivley et al., 2019). The discrimination of male dairy surplus calves with respect to inadequate neonatal care results in high morbidity and mortality rates (Reed et al., 2022). In contrast to recent examinations on farms and during transportation to calf raisers or abattoirs (Boyle and Mee, 2021; Creutzinger et al., 2021), processing plants for animal byproducts have been widely neglected as potential sources of information hinting at deficient animal welfare. The few existing reports on the condition of livestock at processing plants for animal byproducts have pointed out that a remarkable number of animals show signs of unnecessary prolonged pain and suffering (i.e., emaciation, decubitus, claw diseases, signs of incorrect killing; Große Beilage, 2017; Lehnert et al., 2022; Prottengeier et al., 2023). However, none of these studies involved calves. Therefore, to the best of our knowledge, our study represents the first attempt to examine the postmortem conditions of calves at processing plants for animal byproducts.

In contrast to the reported situation in swine and adult cattle (Große Beilage, 2017; Lehnert et al., 2022; Prottengeier et al., 2023), we did not find a high number of calves with signs of poor animal welfare on farms. However, this might be related to general production chain practices in Germany. Most surplus calves born in Germany are sold to the Netherlands and Spain, but are also resold in African countries (Förster, 2021). In contrast to transportation practices in other countries, the transportation of calves is only permitted after the 28th day of life (Bundesministerium der Justiz, 2024a). Therefore, only animals younger than 28 d, originating from smaller holdings or national calf raisers, end up at national processing plants for animal byproducts. We hypothesized that examinations may result in higher numbers of welfare-related findings if they were performed in the aforementioned target countries, because the general condition of sold surplus calves is suboptimal. This factor results in high mortality rates after transportation to the target countries (Creutzinger et al., 2021).

The same reason may account for the nearly even distribution of male and female calves in the present study. Contrary to our hypothesis that male calves would represent the majority of calves in the examined processing plants, we observed no significant sex differences in animal counts.

In nonscientific media and reports originating from animal welfare organizations (Bodderas and Graw, 2019), the illegal killing of neonatal calves is propagated for financial reasons. In the European Union, the identification and registration of bovine animals is mandatory, based on regulation EC no. 1760/2000 (statutory), after the seventh day of life (Bundesministerium der Justiz, 2024b). The time interval between birth and the seventh day of life is referred to as the gray area, during which calves that are born alive may be killed illegally and declared “stillborn” for disposal at local processing plants. For example, Canadian surveys report that ∼5% of dairy producers kill their male calves at birth, and ∼30% use blunt force trauma as a method of calf euthanasia (Hendricks et al., 2022). The findings of our study contradict this suspicion because we could not verify signs of illegal killing in the examined calves at the 2 processing plants. However, we were unable to distinguish between dead calves and euthanized calves in all cases. By contrast, calves that had captive bolt marks with exsanguination on one side and calves with amniotic fluid in the trachea were categorized as legally killed and stillborn, respectively. Calves with no exterior signs of violence or injection marks may not be classified as dead or killed. Nevertheless, 73.9% of calves older than 3 wk had no ear tags, indicating that those animals were at least not registered. This might point toward high mortality rates in single farms or indicate a general deficit in registration practices on farms and should be further elucidated.

The rather high percentage (12.4%) of stillborn calves in our study suggests either a high incidence of abortion or a poor performance of obstetric care and monitoring on the farms. However, further examinations would be necessary to reveal underlying causes. To improve periparturient care on the farms, training and further education of farmers regarding obstetrics and early monitoring of pregnant cows may be worthwhile and are supposed to have the potential of reducing mortality rates. General obstetric care was deemed sufficient, as no signs of forced obstetric assistance, such as fractures of the distal carpus or tarsus or the proximal tibia or femur, were observed.

In line with the proposed use of animal-based measures in slaughterhouses to detect lung lesions as signs of respiratory disease (Nielsen et al., 2023), we examined tracheal discharge in our study. In 6.2% of all calves, suppurative discharge indicated chronic bronchopneumonia, which is one of the most important problems in calves older than 30 d of age (McGuirk, 2008). The percentage of bronchopneumonia (6.2%) in our study was rather low, compared with 21.3% of pneumonia cases in preweaning and 50.4% in weaned heifer deaths (McGuirk, 2008). The incidence of bronchopneumonia in our study was also below the suggested on-farm incidence of bronchopneumonia, which should not exceed 10% (McGuirk 2008).

In contrast to studies on adult cattle (Lehnert et al., 2022), we could not trace any claw pathology or decubitus. Cases of chronic arthritis were present in 0.9% of the calves examined in our study, compared with 3.6% in adult cattle (Lehnert et al., 2022). The notably higher mechanical load on the locomotion system in adult animals might explain the differences in the frequency of occurrence. Additionally, the difference in housing conditions between cows and calves (slatted floors versus straw bedding, respectively) may contribute to a higher percentage of adult animals developing decubitus.

The limitations of our study include the restriction of our examinations to only 2 processing plants. Nonetheless, the total animal count of 981 calves was regarded as sufficient to evaluate the presence and ratio of welfare-associated abnormal findings in the calf carcasses. Intense necropsies with opening of the thorax or abdomen or histopathological examinations were also not performed for technical reasons and because we intended to not interfere with the routine working chains of the processing plants. An eligible further modality of examination of processing plants for animal byproducts would be to collect data concerning the exact age of the fallen animals, possible back-tracing of the calves to their farms of origin, and the realization of farm visits in cases in which abnormal findings may be back-traced to the same farm.

In contrast to the published situation in pigs, we could not determine any evidence of improper killing or illnesses that led to prolonged suffering and unnecessary pain before death in the 2 examined processing plants for animal byproducts. Therefore, poor animal welfare was not an inherent problem in this system. Simultaneously, individual cases of chronically ill animals were identified and would have led to a complaint, similar to the situation at abattoirs, if many such cases had originated from the same farm. Future feasibility studies may evaluate a back-tracing system in suspected cases. However, no fundamental need existed for routine testing of animal carcasses at processing plants for animal byproducts, at least in the catchment areas of the plants investigated.
